# A deep dive into addressing obsolescence in product design: A review

**DOI:** 10.1016/j.heliyon.2023.e21856

**Published:** 2023-11-04

**Authors:** Lesly Sierra-Fontalvo, Arturo Gonzalez-Quiroga, Jaime A. Mesa

**Affiliations:** a GIMYP Research Unit, Department of Mechanical Engineering, Universidad del Norte, Km 5 Vía Puerto Colombia, Barranquilla, 080001, Colombia; b UREMA Research Unit, Department of Mechanical Engineering, Universidad del Norte, Km 5 Vía Puerto Colombia, Barranquilla, 080001, Colombia

**Keywords:** Design attributes, Durable products, Product lifecycles, Sustainable design, Consumer behavior, Modularity

## Abstract

In today's fast-paced world, products are constantly replaced by newer and more advanced versions. While some products become outdated due to natural causes such as wear and tear or technological advancements, others are strategically designed with a predetermined shelf life to encourage rapid product turnover. Obsolescence is an essential issue in product design because of its impact on product life, efficiency, and sustainability. Although there are approaches to map and measure possible product obsolescence scenarios, it remains a challenge to quantify and diagnose a product's or component's obsolescence potential based on its design attributes. Therefore, this article aims to analyze the existing literature on obsolescence from a product design perspective. It covers its application in methodological design strategies, metrics for measuring obsolescence from early design stages, and identifying understudied research topics, challenges, and trends. On August 15, 2023, a total of 221 articles published between 1983 and 2023 on SCOPUS, Web of Science, and Google Scholar were selected and analyzed using a content-based research approach encompassing three main aspects: objectives and methodologies, strategies and design phases, and metrics for obsolescence analysis. As main findings, this literature review identified several methodological design approaches aimed at resisting and postponing obsolescence, mainly divided into designing long-life products and extending product life. Nevertheless, this study found no formal identification of product design attributes related to the different types of obsolescence, and obsolescence forecasting metrics have focused on defining whether the scenario happens but do not consider what type of obsolescence the product may exhibit. Consequently, it can be challenging to determine the most effective design strategy to reduce obsolescence. This study has limitations, including the potential for researcher bias to affect the systematization of the information.

## Graphical abstract

**Figure:**
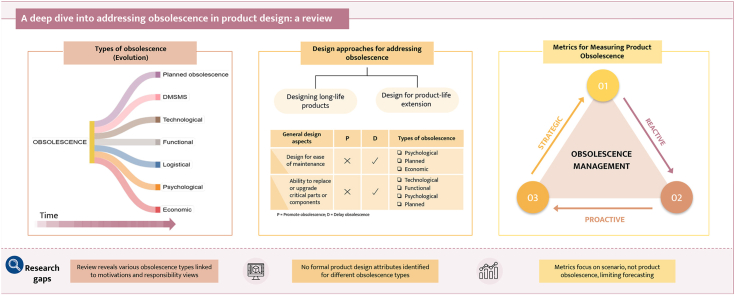

## 1 Introduction

Product obsolescence poses significant environmental and social challenges worldwide. This term refers to the state of being outdated or no longer in use, and it can denote products or technology that are no longer relevant or useful in the current context. The rapid acceleration of consumerism, often referred to as the “Throw-Away Society,” has serious consequences [1,2]. The production and consumption of new products, coupled with the disposal of older ones, contribute to the depletion of non-renewable resources and pollution of the biosphere [[2], [3], [4], [5]].

In the past, manufacturing companies focused on designing high-quality, reliable, and long-lasting products [6,7]. However, with technological advances and the introduction of new applications and features, manufacturers have adopted new strategies to increase sales and meet customer needs [1,[8], [9], [10]]. As a result, product lifetimes have shortened, and mass production has made components obsolete even when still functional [2,4,11]. A prime example is the electronics industry, where complex electronic systems, product manufacturers, and users are particularly susceptible to obsolescence due to the rapid pace of technological advancements, leading to new and improved products entering the market frequently.

Obsolescence is an issue that all products or systems face at a certain point in their lifecycle, and product design plays a crucial role in minimizing or promoting it. For example, products designed for long lifetimes, like aircraft, often outlast the components inside them. This problem represents a barrier to extending a product's life, as replacing outdated parts can be expensive or even impossible [[12], [13], [14], [15]]. A product's life properties and a large percentage of the total cost can be determined during the design stages. In fact, a designer can decide how to reduce emissions, natural resource consumption, waste generated throughout a product lifecycle, and characteristics that lead to obsolescence [4,13,16,17]. Therefore, understanding product obsolescence can help keep products in use, reducing the need for new manufacturing and waste generation. This can also encourage companies to develop more durable products and circular business models [18].

A Circular Economy is a framework that aims to extend the value of resources, materials, and products within the economy while reducing waste [19]. Transitioning from a linear to a circular economic model has been explored from an industrial design perspective. Circular product design involves two key aspects: designing for product integrity to prevent and reverse obsolescence at the product and component level and designing for recycling to prevent and reverse obsolescence at the material level [16]. Researchers have proposed different methods and tools to extend product lifespan, including business models, product design strategies, metrics, and obsolescence risk assessments. This work aims at reviewing and discussing these design strategies and obsolescence risk metrics from a product design perspective.

A literature search yielded two recent review articles focused on obsolescence: Sonego (2022) [20] and Rivera and Lallmahomed (2016) [11]. [20] analyzed the barriers and motivations for repairing electronic products, focusing on consumer behavior and the environmental benefit of extending the product lifecycle through repair. The article discusses government and non-governmental initiatives to promote repair and addresses technological obsolescence. However, it does not deeply into other important aspects of repairing electronic products, such as technical or economic challenges arising during the repair process and how to address them from the design stage. On the other hand [11], explored planned obsolescence (PO) and its environmental impact during a product's useful life. Their study aimed to highlight the role of product lifetime in product obsolescence and identify ways to integrate product lifetime into the design process and business models. However, it does not discuss the design strategies that can promote extending product lifespan. Despite these reviews, there is a research gap in design methodologies and metrics for addressing obsolescence from the early design stages. Therefore, this article aims to map product design attributes and methodological approaches regarding product obsolescence to:

i) Identify the most relevant product design attributes that impact product obsolescence either by promoting or delaying it. This will enable the creation of products better adapted to customer needs.
ii) Characterize design methodological approaches that address product obsolescence. This will provide a theoretical basis for principles and strategies and identify opportunities for designing sustainable products.
iii) Identify and analyze existing metrics or indicators for measuring product obsolescence. This will enable more accurate assessments of the impact of different design attributes on product lifespan and obsolescence.

The remainder of this paper is structured as follows. First, section 2 describes the methodology used for the literature review, including the snowballing technique and the inclusion criteria for selecting articles. Next, section 3 presents the research results, illustrating the evolution of the “obsolescence” concept and its application in product design. Section 4 discusses the main findings and describes the research gap. Finally, we conclude with final remarks on the contribution of this research, its limitation, and promising areas for further investigation.

## 2 Methodology

This study employs a two-step approach: (1) conducting a literature search on product obsolescence using specific criteria to select relevant works, and (2) reviewing and assessing obsolescence in product design based on the selected papers. The following subsections describe the methodology used for the literature search and the analysis of the selected works.

### 2.1 Literature search and selection criteria

The research was based on a structured literature review. First, an exploratory search was conducted between November 16 (2022) and August 15, 2023, using Scopus and Web of Science (WOS). The research protocol involves the following keywords: (“Product design” OR “design strategies” OR “design principles” OR “design guidelines” OR “sustainable design”) AND (obsolescence OR “durable product” OR lifespan OR “extended lifespan”), applied to the title, abstract, and author's keywords fields. This search yielded 877 articles: 624 from Scopus and 253 from WOS.

A Google Scholar search was also conducted using the exact keywords and organizing by relevance. Due to the large number of documents and to limit the search to more current research relevant to the research topic, the search was limited to papers published between 2000 and 2023 from the first ten pages. This search yielded 100 articles.

The total number of articles from the three databases was 977. However, results showed some unrelated topics, such as structural design, building obsolescence, professional obsolescence, and lithium-ion batteries, among others. Therefore, the keywords identified in SCOPUS and WOS were analyzed to determine unrelated terms and generate a refined query on obsolescence in product design. From this, the search equation was extended as follows: (“Product design” OR “design strategies” OR “design principles” OR “design guidelines” OR “sustainable design”) AND (obsolescence OR “durable product” OR lifespan OR “extended lifespan”) AND NOT (concrete OR building OR cement* OR corrosi* OR cataly* OR “architectural design” OR “structural design”) AND NOT (electrode* OR anode* OR “professional obsolescence” OR dendrites OR capacitor OR immune* OR medic* OR human*). The number of articles was reduced to 654.

**Fig. 1:**
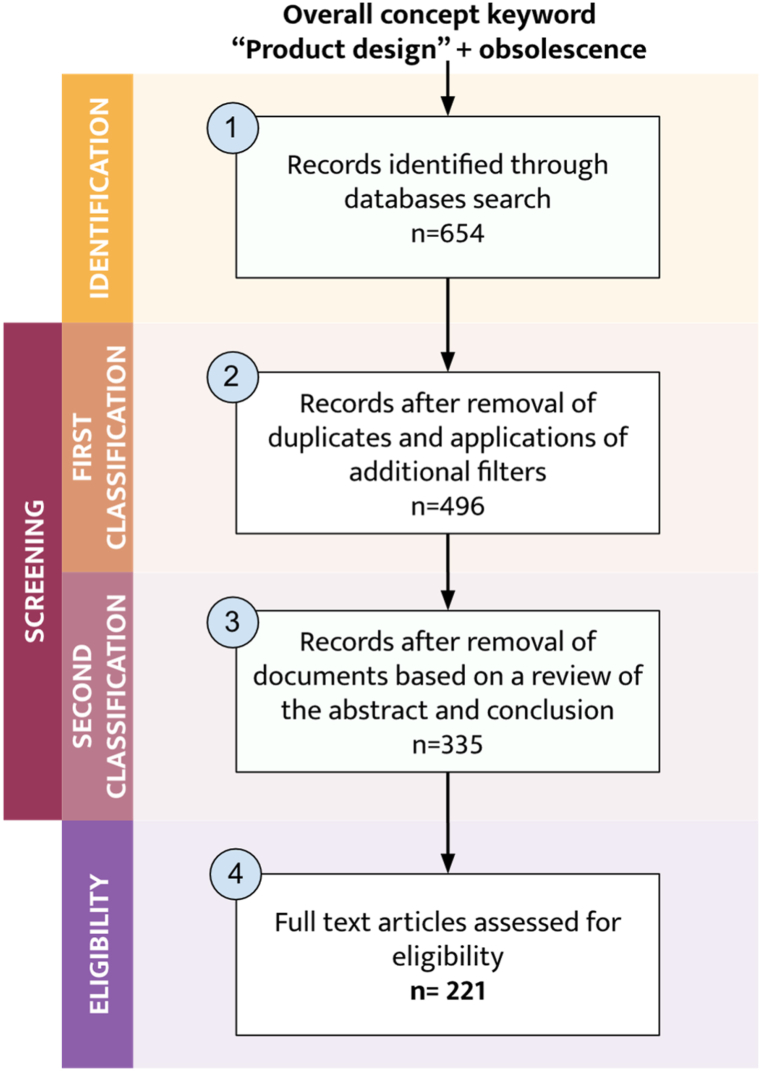
Flow chart of the literature search and selection of articles.

Then, we filtered the articles by type (original articles, literature reviews, book chapters, and proceedings) and language (English). After removing duplicates, the number of reports was 496 (401 from Scopus, 37 from WOS, and 58 from Google Scholar). A final evaluation of the abstract and conclusions made screening the 496 resulting articles possible, after which 335 articles reached the eligibility and inclusion phase. Inclusion criteria for the eligibility phase were papers on types of obsolescence, design methodologies, indicators, trends, evolution, drivers and barriers, and global engineering or product design context. The final set comprised 221 eligible papers. It is important to note that a few non-peer-reviewed articles were included because obsolescence is a new area of research, and its relationship with sustainability has not been extensively addressed. Fig. 1 illustrates the methodological steps undertaken.

### 2.2 Analysis of selected works

The three authors of this paper independently chose the studies based on the inclusion criteria stated in the previous subsection. They classified the studies in two stages. Initially, the investigators compared their results and selected the articles they agreed on. Subsequently, they excluded articles that did not match the inclusion and exclusion criteria and organized the information in an Excel sheet. The content of the selected studies was mapped by extracting information into two categories: general and focus areas. This categorization enabled a systematic analysis of the information from general to specific. The general category included information such as author, year of publication, article title, and context. This category covered the analysis of literature trends, the relevance of the concept of obsolescence over the years, and the objective and actors involved. It also analyzed the number of selected documents financed by private interests to assess the risk of selective bias in the information.

The second category involved a content-based analysis, including the examination of methodologies, design stages, and design strategies. This category revealed the leading research topics and research gaps related to product obsolescence. To synthesize the results, the papers were categorized according to a thematic analysis into three main groups: evolution of the concept of obsolescence, design approaches to address obsolescence and metrics for measuring product obsolescence. This provided a holistic picture of obsolescence from a product design perspective. The following section will present the results obtained from a thematic literature analysis.

## 3 Product obsolescence

**Fig. 2:**
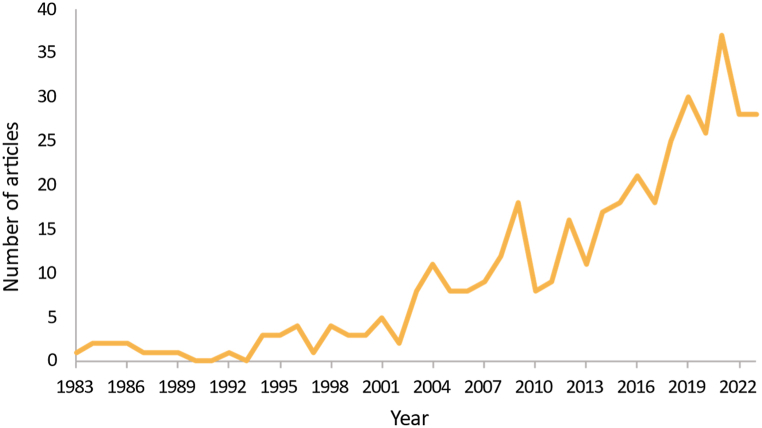
Time evolution of research papers related to product obsolescence published since 1983.

**Fig. 3:**
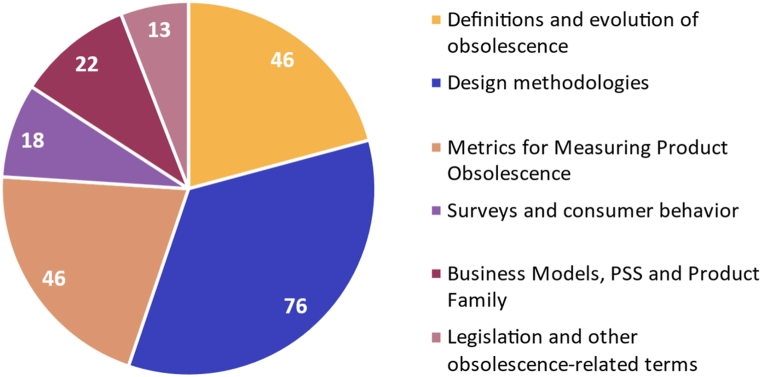
Thematic analysis of selected articles in relation to product obsolescence.

The review covers four decades of research from 1983 to 2023. According to the extracted general data, the most relevant sources identified are the Journal of Cleaner Production (21), Proceedings of the ASME Design Engineering Technical Conference (14), Sustainability (13), and Procedia CIRP (11). Fig. 2 shows the increase in the number of publications on product obsolescence, with an annual growth rate of around 8.69 %. Out of the 221 scientific articles reviewed, 34 % focused on methodological design approaches for addressing obsolescence, 21 % provided metrics for the risk of obsolescence, 21 % discussed the types of obsolescence, and the remaining 24 % covered topics such as surveys, consumer behavior, Product Family, business models, Product Service Systems (PSS), and other obsolescence-related terms (durability, circular economy, among others). Fig. 3 displays the number of documents for each topic. Similarly, no entity did not fund 61 % of the selected writings, 31 % were funded by national or university research and innovation programs, and only 8 % were funded by private entities.

Based on the results of the thematic analysis, the following subsections describe: I) the evolution of the concept of obsolescence characterized by the definition of the types of obsolescence and the perception of responsibility held by consumers and manufacturers, II) the design methodologies focused on resisting and postponing obsolescence, and III) the metrics for assessing the risk of product obsolescence characterized by three action approaches: reactive, proactive, and strategic management. The study excluded topics related to consumer surveys, laws and legislations, and business models (Fig. 3) since this work aims to identify how obsolescence is approached from the design phases and the tools proposed by designers.

### 3.1 Evolution of product obsolescence: classification and perceptions of responsibility

Obsolescence has been the subject of various research projects, and over time, different types of obsolescence have been defined. The term “obsolescence” refers to the state of a material, part, device, software, or product when it is no longer maintained or required by the user, even if it still functions [2,17,21,22]. According to Proske (2019), obsolescence is not a neutral description of a particular natural state of an object. Instead, it refers to a process where something is actively discarded or considered “out of date” [23].

Obsolescence has existed for centuries, but this term became popularized in 1932 by real estate investor Bernard London. He postulated that the “production-consumption-work” cycle must be maintained at all costs to promote the economy (London, 1933, as cited in Paricio et al., 2019). This type of obsolescence was called “product failure.” Similarly, in 1960, Packard [8,25] divided obsolescence into function, quality, and desirability. He described that function obsolescence occurs when a product is replaced by a new product that performs the same function better. Quality obsolescence occurs when a product is intentionally designed to break down, and desirability obsolescence occurs when a product is replaced due to changes in styling or other factors that make it seem less desirable [26].

Alternately, Cooper (2004) categorized obsolescence into absolute and relative groups. Absolute obsolescence includes material wear, technical causes or lack of repair options, and spare parts or components shortage. Relative obsolescence results from changes in consumer requirements that promote the replacing a functional product and is divided into three categories: psychological, economic, and technological [8]. On the other hand, Amoah (2017) identified four types of obsolescence: involuntary, which occurs regardless of whether the customer or the manufacturer wants to change the product [22]; voluntary, when the user/manufacturer allows the technology to become obsolete; expected, when stakeholders know that support services will be discontinued or become obsolete; and unexpected, which refer to sudden changes in the position of the original manufacturer [27]. In addition, Proske [28] introduced two additional types: accepted obsolescence, which occurs due to cost and time pressure and marketing strategies that lead manufacturers to use low-quality materials and components, and obligatory obsolescence, which occurs due to regulations. Postponement obsolescence is another type identified by Amolo [29], referring to when a company adds technological features to one of its products but not all. This type is most common among car manufacturers. For example, Mercedes Benz installs an integrated sensor that helps detect driver fatigue or drowsiness in Model S cars, but not in its other models [29].

**Fig. 4:**
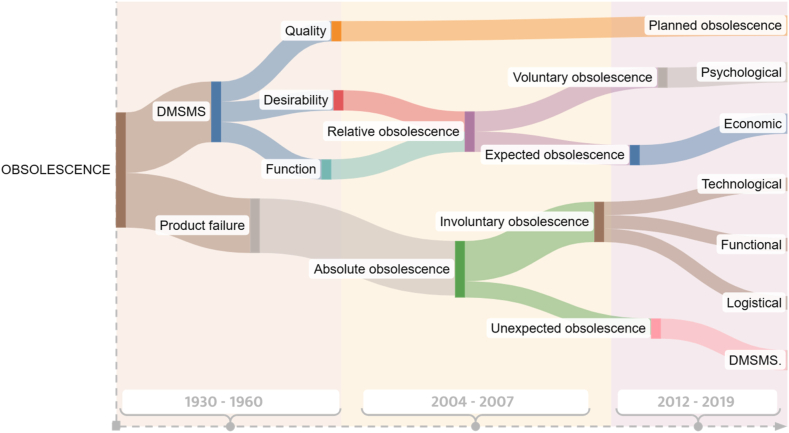
Sankey diagram summarizing the evolution of the different classifications of the term obsolescence based on an analysis of definitions. Each branch refers to how, over the years, various subdivisions have broken down into more specific concepts to define obsolescence depending on the context in which it is presented. DMSMS: Diminishing of manufacturing sources and material shortages.

Despite the emergence of new types of obsolescence, the most mentioned in the literature are planned obsolescence, technological, functional, psychological, economic obsolescence, and diminishing manufacturing sources and material shortages (DMSMS) [16,22,30,31]. Table 1 describes the most common types of obsolescence found in the literature and Fig. 4 shows the evolution of the “obsolescence” concept.

**Table 1:** Definitions and types of obsolescence. Based on [8,22,30].

Type	Definitions	Example
Technological obsolescence	This refers to the rapid obsolescence of electronic and technological products due to the fast pace of technological advancement and the introduction of new products and solutions in the market. Products that were once considered state-of-the-art quickly become outdated and incompatible with the latest technologies and applications.	The evolution of television technology has rendered older models obsolete. As a result, flat-screen TVs with advanced LED, LCD, or plasma technology have become the new standard in home entertainment.
Functional/incompatibility obsolescence	This occurs when the specific requirements of a product change, causing its performance or reliability to become outdated. This term is also related to the inability of a device to support software updates due to a lack of add-on features.	An older smartphone may become obsolete as it is no longer compatible with the latest software and application updates. This limits its functionality and usefulness compared to newer models.
Psychological obsolescence	This appears when functional products are replaced or used shorter than their potential lifespan. This may happen when a loses its aesthetic appeal due to the introduction of new design trends or the users' desire for novelty.	When a brand releases a new product version that includes minimal improvements but markets it as a significant upgrade. This new version is then perceived by society as “state of the art”, rendering the previous version obsolete.
Economic obsolescence	This occurs when it is no longer economically viable to continue using a product. This may happen when the cost of consumables, maintenance, and repair become prohibitively high compared to the relatively low cost of purchasing a new product.	A printer may become obsolete even if it is in good condition. This can happen when the cost of ink cartridges and maintenance exceeds the cost of a new printer.
Diminishing manufacturing sources and material shortages (DMSMS)/indirect/logical	This happens when components, materials, or technologies used in a product are no longer available in the market due to discontinued manufacturing or lack of supply. DMSMS can result in supply chain disruptions, production delays, and increased costs.	When a company produces a product using specific components or materials that eventually become unavailable in the market. As a result, older products may not be upgradeable or repairable.
Planned obsolescence	This refers to designing and producing products with a predetermined lifespan or period of usefulness. This means the product becomes obsolete or unusable after a certain time, prompting consumers to purchase a replacement.	The use of batteries in electronic devices that are designed to last for a limited number of charge and discharge cycles.

The literature review also reveals differences and subcategories in the types of obsolescence related to motivations and perceptions of responsibility. For example, in the conventional linear economy model, planned obsolescence (PO) is associated with the manufacturer as the sole responsible party [16,32,33]. Proske [28] defined PO as the “deliberate shortening of the product lifetime by the manufacturer.” Some authors view PO as a tool to recover market share and contribute to technological progress and innovation [2,25,34]. In contrast, others highlight its adverse effects, such as pollution, increased waste, and depletion of natural resources [2,35].

On the other hand, psychological obsolescence focuses on consumer behavior as a contributing factor to increasing waste. This type of obsolescence is divided into emotional, style, aesthetic, cosmetic, and social [32,36]. In general, psychological obsolescence, also named perceived obsolescence, appears when functional products are replaced or used for less time than they could be used [37]. For example, the fast fashion movement and trends change promote that older products become less attractive and discarded for style obsolescence [29]. Moreover, it reduces the emotional ties that consumers have with their products driving to increase in users’ desire for new products [16,38].

### 3.2 Design approaches for addressing obsolescence

A product rarely faces all the forms of obsolescence simultaneously. Despite the global product facing psychological or technological obsolescence, many components may still function [16]. Consequently, researchers and product designers have begun exploring methodological approaches to managing obsolescence [39], resulting in various design strategies that ensure prolonged usage. For instance, Guiltinan (2009) identified two primary mechanisms of obsolescence: physical and technological. Physical obsolescence includes design for limited repair, limited functional life, and aesthetics [40]. On the other hand, technological obsolescence encompasses design for fashion or style and design for functional enhancement [2].

Furthermore, reducing obsolescence correlates closely with CE strategies. According to Proske (2019), CE strategies can be “translated” into product design strategies for long-lasting products. These strategies include the design for reliability and durability, repair and maintenance, standardization and compatibility, upgradability, variability, and product attachment [41]. Likewise, Shevchenko (2022) characterizes circular products and proposes to consider CE-related product attributes as (I) products that contribute to closing the loop (recyclable products and products with recycled content, and (ii) products that contribute to slowing the loop (reusable products and products with reused content). This study presents that If a product can implement multiple strategies simultaneously, it has a high circular impact and can be positioned in a “Closing–Slowing Future–Past’ quadrant” (CSFP). The CSFP quadrant model categorizes products based on their sustainability: Quadrant I - Products with recycled content from the past, Quadrant II - Recyclable products for the future, Quadrant III - Reusable products for the future, and Quadrant IV - Products with reused content from the past [42].

**Fig. 5:**
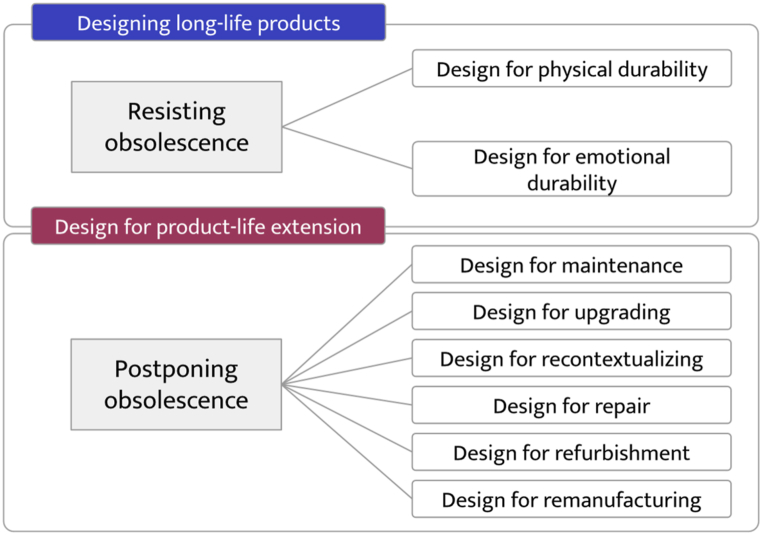
Typologies of design approaches for product integrity.

From a global design perspective, the review shows that different design strategies are divided into two borad approaches: designing for long-life products and designing product-life extension [38,43]. Fig. 5 presents general strategies for durable design found in the literature. Each one of the methodological approaches show in Fig. 5 is described below.

#### 3.2.1 Design for long-life products

According to Bocken (2016), long-life product design is supported by design for attachment and trust, reliability, and physical durability [43]. In this approach, design for reliability is related to designing for a “high likelihood that a product will operate throughout a specified period without experiencing a chargeable failure” (Bocken et al., 2016, p3). In this sense, an essential phase of the design process for long-life products is the selection of materials. According to Lilley [44], considering materials’ changes during their lifetime can help guide products toward sustainable use, care, and maintenance patterns. As a result, some authors propose material change as a tool in design. For instance, Frahm [36] investigated the tools used for material selection in different companies and found that prototyping, accelerated aging testing, material samples, experience, data sheet analysis, and trend detection are the most common in the industry [36]. Nevertheless, these material selection tools must adequately predict how the material will behave during its use cycle. For example, the material samples are usually presented in pristine conditions or an unquantified state of degradation (Lilley et al., 2019).

Besides, design for attachment and trust is related to products with a high user connection that incentivizes them to keep their possessions for an extended period [38,43]. According to Hagedorn (2021), the greater a consumer's attachment to a product, the greater their willingness to repair it [37]. Thus, increased product lifespans require consumers to keep their possessions longer without discarding them, and the drivers for increased product attachment are diverse. For example, Mulet (2022) investigated the attachment strategies applied to household appliances like toasters, vacuum cleaners, and personal care appliances using “The emotional durability design nine” tool. The results showed that the most frequently used strategies are related to design for animacy, variability and modularity, and connection or community belonging [38]. Similarly, Hagedorn (2021) investigates the impact of aesthetic mass customization. He found that consumers use individualized products longer, creating product attachment and brand loyalty [37].

#### 3.2.2 Design for product life extension

The second approach refers to lifecycle extension. This strategy is directly related to the main ideas of a CE and the hierarchy of the 9Rs [28,38,43]. Thus, depending on the applied design strategy, a product's useful life can be extended in three forms: products, components, or materials [45]. For this approach, strategies such as maintenance and repair, upgradability and adaptability, standardization, compatibility, and disassemblability design were found [25,43,45]. These concepts share a common characteristic: they all involve some form of alteration in the relationship between the device owner and the device. This relationship could include transferring ownership of the device, repairing or recycling it, or changing how people use it by upgrading it [46].

For this reason, consumers play a critical role in product design since their decision defines the end of a product's useful life. According to Sonego (2022), to promote longer product lifecycles through design for repair, it is essential to consider the factors that hinder product repair from the viewpoint of consumers. Among the different barriers encountered are cost, time, lack of information, quality of repair services, and negative prior experiences [47,48].

Also, numerous methodological design approaches have been developed to increase efficiency at the design stage. These methodologies have been grouped into a concept called “Design for X (DfX)” [49,50]. According to Ref. [49], DfX can be divided into two subjects: design for efficiency, which includes strategies based on reducing the cost and lead time of a product while sustaining or improving its quality, and green design, which aims to minimize negative environmental impact throughout a product's life cycle by incorporating natural systems [49]. Table 2 summarizes the most common design for x methodologies in the literature.

**Table 2:** List of different product design strategies for product life extension based on [[51], [52], [53], [54]].

Category	Scope	Design for X - Strategies		Objective
Design for efficiency	Product Product	DfM	Design for manufacturing	Conserve resources during product fabrication while facilitating efficient and swift, high-quality production.
		DfA	Design for assembly	Improve product assembly by simplifying the process and reducing harm and wear.
		DfD	Design for disassembly	Simplify disassembly for quicker, complete repair, reducing waste and resource use and aligning with environmental concerns.
		DfRem	Design for remanufacture	Design to enable disassembly, assembly, cleaning, testing, repair, and replacement.
	Product and system	DfQ	Design for quality	Develop a design that reduces manufacturing defects and meets customer expectations.
Green design	Product	DfE	Design for environment	Assess the environmental safety and health aspects and "close the loop".
		DfRe	Design for Recycling	Enhance the potential for material recovery, encourage the use of recyclable materials, facilitate material identification, and design to maximize the utilization of recyclable materials while minimizing material diversity.
		DfS	Design for sustainability	Design to consider the three dimensions of sustainability: economy, ecology, and equity
	Product and system	DfLC	Design for lifecycle	Assess overall resources consumed and pollution generated in the product lifecycle and design to reduce life cycle cost
		DfMLC	Design for Multiple Lifecycles	Dividing a product's life into multiple cycles to reuse and upgrade some parts while replacing others to prolong its useful life.

Product modularization is among the most common designs for X approaches. Design for modularity enables the independent replacement of subsystems, allowing customers to replace only the obsolete subsystems while keeping the remaining parts of the product intact [[55], [56], [57]]. This approach aims to optimize operations relative to the product's end-of-life, such as reusability, recycling, maintainability, and technological updating [58]. Since only some subsystems become obsolete, modularity offers design flexibility and reduces production and disposal costs [41,56]. Umeda (2008) suggested that a modular structure differs according to the applied lifecycle options. For instance, to enhance recyclability, materials that can be recycled without separation should be grouped in the same module [58]. Conversely, some researchers believe that modularity accelerates the introduction and replacement of products at the modular subsystem and product levels. As a result, the disposal and constant upgrading of modules increase the environmental burden [55,56].

For the above, designers significantly impact product obsolescence due to their involvement in the early stages of product development. Therefore, identifying the product design attributes that affect obsolescence from early design stages will enable the creation of products adapted to customer needs and support closed-loop supply chains.

#### 3.2.3 Design parameters

According to the previous section, obsolescence takes different forms depending on the context, and designing against it can contribute to sustainability during product design. For this reason, researchers have focused on identifying the parameters or design attributes that can promote or delay obsolescence according to product functions and customer needs. For instance, Blijlevens (2009) researched appearance attributes that customers use to distinguish durable products. This research aimed to provide knowledge on the consumer perception of product appearances and identify the aesthetic qualities that distinguish durable products, such as color, shape, and sense of modernity [59]. Likewise, Joustra (2021) identified four clusters of design attributes for composite products: handling and rework, product architecture, specifications, and traceability [60].

From a design research perspective, the design guidelines proposed in subsection 3.2 can be applied and integrated to identify general design attributes. For example, Zhang (2019) investigated the challenges of identifying design factors for the DfRem method. The article proposes a process to identify the characteristics from the failure modes information. As a result, it obtained that ease of disassembly and separation, ease of access, ease of cleaning, and ease of handling are some of the attributes of design applicable for engine crankshafts for remanufacturing [52]. Table 3 summarizes general design aspects that tend to promote or reduce product obsolescence in the literature. It is suggested that every product should be evaluated, particularly in the design stages, against each mode of obsolescence to determine which will likely be its weakest link.

**Table 3:** General design aspects that promote or delaying product obsolescence based on [38,47,61].

General design aspects	Technological	Functional	Psychological	Economic	Planned	DMSMS	Relationships
Use trendy designs or fast-changing trends	P		P		P		3/6
Designing a product that stimulates feelings of attachment	D		D		D		3/6
Include additional features to the primary function of the product		D	D		D		3/6
Incorporate technologies that are not compatible with other products	P	P	P		P	P	5/6
Repairability	P	P		P	P		4/6
Low software upgrade capability		P	P		P		3/6
Ability to replace or upgrade critical parts or components.	D	D	D		D	D	5/6
Designing a product resistant to degradation over time			D		D		2/6
Design for multiple lifetimes					D	D	2/6
Material quality			D		D	D	3/6
Design for ease of maintenance			D	D	D	D	4/6

P = Promote obsolescence; D = Delay obsolescence.

### 3.3 Metrics for measuring product obsolescence

All products experience obsolescence at some point in their useful life. While functional obsolescence can be easily predicted in the design phase, other types of obsolescence are more challenging to predict due to consumer behavior, market trends, and technological advances [16,62]. Different obsolescence management approaches consist of methods, tools, and processes for detecting and mitigating obsolescence at the component or system level. As a result, obsolescence management has been categorized into three kinds: reactive, proactive, and strategic management [12,17,22,63,64].

Reactive obsolescence management is one of the most common approaches to addressing obsolescence problems [13]. This kind of management consists of determining an appropriate and immediate resolution for an obsolete component [17,22,65]. This approach is usually found in a sustainment-dominated system such as aircraft and ships. Some obsolescence mitigation strategies include lifetime or last-time buys, part substitution, uprating, and redesign [13,17,63]. On the contrary, proactive management involves a combination of different measures to prevent or minimize the effects of obsolescence. Some proactive approaches are forecasting methodologies, such as evaluating the risk of obsolescence, BOM management, material risk index, integrating obsolescence in the traditional analysis of failure, and identifying obsolete functions or critical components [13,17,22,64,65]. Table 4 shows some current methods for forecasting obsolescence found in the literature.

**Table 4:** Obsolescence management strategies.

Authors	Metric	Parameters involved	Focused on	Case study
[67]	Life cycle curve	Device/technology group, primary and secondary attributes of parts, number of sources, sales data of the primary attribute	Forecast life cycle stage and the years to obsolescence of electronic parts	16 M DRAM
[14]	Component Obsolescence Risk Assessment	Technological characteristics	Predict new product introduction dates based on a linear regression model	Computer processors
[68]	Obsolescence risk forecasting	Technical specifications of each product.	Predict the label of active or discontinued of a product using machine learning.	Smartphones
[69]	LCA - Environmental Impacts Assessment	Consumer repair behavior, deterioration process of components	Assess the environmental impact associated with the life cycle of a consumer electronic	Laptop
[7]	Cumulative customer satisfaction over time for evaluating products	Expected customer satisfaction, sustained usage rate	Predict the impact of an obsolescence/DMSMS	Smartphones
[70]	Design Integrity Risk	Principles of design of each part (resisting, postponing, and reversing obsolescence)	Obsolescence and recyclability integration in a Circularity Impact and Failure Analysis	Bicycle V-brake system
[71]	Obsolescence index	Product operating data (performance data) Customer requirements Desired level of a product's functional performance	Identify obsolete functions of a system	Smartphone
[13]	Obsolescence risk assessment	Technology readiness levels (TRLs) for the likelihood Number of interfaces within system elements	Identify critical components with the highest risk of obsolescence	Knock detection functionality for gasoline engine management systems
[5]	LCA Environmental Impacts Assessment	Use-time Reference Flow	Evaluate anti-obsolescence strategies	Fairphone 3
[72]	Obsolescence risk forecasting	Identify the key product features for obsolescence using the ELECTRE I method	Calculates the obsolescence probability based on radial basis function neural network.	Mobile Phone

According to Sandborn (2008), strategic management uses obsolescence data, technology forecasting, logistics data, and business trends to assist strategic planning and lifecycle optimization. Strategic management of obsolescence focuses on minimizing the risk of obsolescence from the early design stages and over the product's lifecycle [17]. This approach involves reactive and proactive strategies [22]. Some of the most common strategic management approaches are material risk indices and design refresh planning (DRP) [63]. DRP is gaining attention since it improves the system's efficiency by allowing plans to update specific technological components when they become obsolete [66].

Another product obsolescence metric found in the literature is life cycle assessment (LCA). This approach does not fit into the previous categories. Nevertheless, it is widely used to provide information about the environmental impact of products and the efficiency of anti-obsolescence strategies [11,41]. An example would be the utilization of LCA to examine various measures to prolong the lifespan of smartphones [5]. This method generally allows for measuring the overall material and resource inputs in product manufacturing. Nonetheless, a limitation of LCA is that it does not consider consumer behavior [11].

In the case of product families, long-lived platforms are prone to obsolescence. Kang (2012) discusses common challenges faced in managing product family obsolescence and how planned platform replacement can help mitigate these challenges. Planned replacement refers to the strategy of replacing obsolete components or systems in a gradual and planned manner rather than making abrupt changes. This strategy involves determining the optimal time to replace an existing platform with a new one, considering factors such as the costs and benefits of a replacement, market trends, and customer demands [73].

## 4 Findings and discussion

The literature review results show an increase in the number of publications regarding obsolescence over the last five years. The growing interest in sustainability has driven the search for more durable and environmentally friendly alternatives. For this reason, research in this field seeks to understand the causes and effects of product obsolescence and to develop strategies for its prevention and sustainable management.

The analysis of the selected works focused on their objectives and methodologies, examining how they related to the existing literature and how successfully they achieved their goals. Out of the 221 scientific articles reviewed, we found three main topics highlighted: concepts and evolution of obsolescence, methodological design approach, and case studies focused on proposing tools and metrics to measure the risk of product obsolescence at the component or system level. The main contributions of each of these topics are discussed below and we propose a research agenda for advancing the field, summarized in Table 5.

**Table 5:** Research gaps and research agenda regarding obsolescence and product design.

Aspect	Research gap	Research agenda
Design approaches for addressing obsolescence	i) Research on defining requirements in the product design process has been limited. More research is necessary to identify product design attributes that impact product obsolescence. ii) There is not a formal identification of product design attributes related to the different types of obsolescence such as technological, functional, psychological economic, DMSMS, and Planned. iii) Little attention has been paid to defining materials as a design strategy. More research is needed to understand the appearance and wear of materials during their life cycle.	i) Developing additional approaches focused on determining the definition of requirements and design attributes that impact product obsolescence (promoting or delaying) ii) Generating formal design rules aimed at delaying obsolescence and slowing the loop of resources iii) Characterizing product attributes or features respect to the different types of obsolescence iv) Developing approaches aimed at defining/selecting materials to achieve product durability from the early design stages considering the product life cycle.
Metrics for measuring product obsolescence	iv) Obsolescence forecasting metrics have focused on defining whether the scenario happens but do not consider what type of obsolescence the product may exhibit. Consequently, it can be challenging to determine the most effective design strategy to reduce obsolescence. v) Most of the research focuses on metrics for electronic products.	v) Developing additional obsolescence assessment tools focused on types of obsolescence. vi) Developing more design methods and indicators to measure, determine and predict product obsolescence in different lifecycle scenarios.
Obsolescence in product families	vi) There is a lack of research oriented to product families and product platforms, particularly when it comes to analyzing different types of obsolescence	vii) Conducting research on obsolescence concerning product families and product platforms, which are commonly employed to develop products across multiple generations and promote the sharing of design tasks, manufacturing processes, and modules

### 4.1 Concepts and evolution of obsolescence

As the first research direction, we focus on analyzing the evolution of the term obsolescence and its different classifications through the years. Although the literature presented in this paper does not establish dates of appearance, it is possible to determine a flow of terms or concepts over time, as illustrated in Fig. 4. The term obsolescence presents its first ramifications and classifications in the early 2000s. Although the term was well known in the fashion industry, in product design, it appeared first after the Great Depression with characters such as Bernard London. During the period, mass production was necessary to promote the economy and job creation, and obsolescence allowed for the constant flow of products and waste. This positive approach to obsolescence allowed different manufacturers to change their market strategies so that the useful life of different products was significantly reduced. An example is what happened with the agreement of light bulb manufacturers to reduce the useful life from 2500 h to 1000 h [11,24].

The common classifications of obsolescence include technological, functional, psychological, economic, planned, or programmed obsolescence and DMSMS. Each classification addresses a specific study area related to technological, economic, and social development. Obsolescence can be seen as a phenomenon that occurs in three areas or levels: technological, psychological, and commercial. Within the technical realm, obsolescence is approached as an inevitable consequence of the rapid acceleration in technological advancement and electronic systems. As new electronic devices appear on the market, the old ones and the products that comprise them begin to be displaced. The constant improvement, related to the desire to innovate on the part of the manufacturer and the consumer, leads to different products losing support in the market, limiting the ability to obtain indispensable parts or systems for a product, which causes the consumer to decide to replace and discard, rather than repair.

Similarly, the psychological sphere is directly related to consumer behavior, conditioned by its social and economic context. This category includes psychological obsolescence in its various classifications, functional and economical. Obsolescence is often presented as the consumer's desire to replace a product, either because of the feeling of updating, aesthetics, the fashion of the moment, or changes in its social context. All products are subject to obsolescence at any point in their life cycle, so addressing changing consumer needs from the design stages becomes an unavoidable task. Finally, the commercial environment goes directly hand in hand with the manufacturer's objectives. Obsolescence should not be viewed as an isolated issue related solely to technological progress or changes in product production. Instead, each of the above classifications is closely related to the other. Some authors even describe each type of obsolescence as a subcategory of planned obsolescence. In this sense, this concept represents a broad topic of study, and different design methodologies and obsolescence forecasting techniques should be addressed to address or minimize it from the early stages of product design.

### 4.2 Design approaches for addressing obsolescence

This paper reviews design methodologies for extending product use time, including long-life product design and design for product life extension. While these strategies are related, they offer different approaches to addressing obsolescence, as shown in Fig. 5. Long-life product design focuses on creating lasting products using durable materials and manufacturing methods that facilitate repair and maintenance. This approach represents the traditional design methodology of physical durability. Within the literature, several authors have emphasized the importance of material selection as a tool for promoting sustainability. However, within the field of product design, this has typically been considered only in terms of physical durability, rather than psychological durability. In this sense, material wear is a primary driver of obsolescence. During a product's use stage, its aesthetics can encourage or discourage a consumer's desire to replace it. Therefore, understanding how materials behave and how their aesthetic wear throughout their life cycle is crucial in the fight against obsolescence. However, this study area has not yet been widely addressed, resulting in a lack of tools for material selection based on material life cycle and natural wear. This represents a research gap in the design of durable products. On the other hand, design for product life extension focuses on promoting the reuse, repair, and upgrading of existing products to prolong their useful life. This methodology focuses on product behavior during use to foster sustainable systems and minimize factors contributing to obsolescence. The design methodologies found are related to CE strategies and have been approached from a design for X perspective. This concept encompasses different methodological approaches to increase efficiency at the design stage, such as design for efficiency and green design. However, more literature should be on this methodology's design parameters. As presented in section 3.2.3, a designer has the responsibility to decide about the useful life of a product from the early stages of design, and the use of this type of methodology allows defining how it will be treated at the end of its useful life, as well as the possible obsolescence that it may present. However, no specific design attributes relate to each methodology, nor are there any general design attributes. In turn, these methodologies have yet to be reviewed, considering obsolescence. The technical, aesthetic, and functional characteristics play an essential role in the obsolescence of a product, either delaying or postponing it, making it inevitable to review the design attributes that have a greater incidence in obsolescence and what type of obsolescence it refers to. A product at risk of functional obsolescence requires different strategies than one with psychological obsolescence. For example, the mechanical components of washing machines do not need to be approached from an aesthetic perspective. However, the door or the casing that covers it must be designed with aesthetics in mind so that it continues to appear functional to the user. Consequently, a second research direction involves identifying design attributes that impact product obsolescence (either promoting or delaying).

### 4.3 Metrics for measuring product obsolescence

**Fig. 6:**
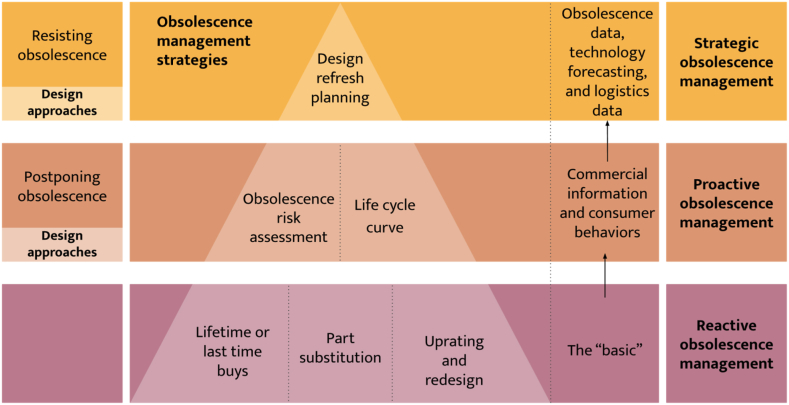
Classification of design methodologies and metrics based on the obsolescence management categories.

While obsolescence is a growing concern due to its impact and universality, much work remains to be done to predict, assess, actively manage, and mitigate it. The literature identifies three approaches to managing obsolescence: reactive, proactive, and strategic. These approaches depend on the importance manufacturers place on obsolescence and available resources. Reactive obsolescence management has been the most common approach. In the past, obsolescence was viewed as an inevitable phenomenon, and the methods to deal with it were reactionary behaviors aimed at avoiding product scrapping. Reactive obsolescence management involves addressing the problem after it has already arisen, such as when a part or component becomes obsolete and needs to be replaced. Consequently, these methods can be very costly over the life of a system, requiring time and resources to implement solutions that may not be available when needed or may not provide adequate parts support over the system's life. This type of obsolescence management represents the lowest level of importance that can be placed on a product, as its goal is to mitigate obsolescence when it occurs rather than minimize it for as long as possible, as shown in Fig. 6.

The main difference between reactive and proactive obsolescence management is the temporal and strategic approaches to address the problem. Proactive management involves anticipating and preventing obsolescence problems rather than reacting to them. Current methods for forecasting obsolescence fall into two broad categories: obsolescence risk and life cycle. Obsolescence risk methods are characterized by calculating a risk index or the probability of a part becoming obsolete. Conversely, lifecycle methods rely on the life cycle curves of a product based on its sales to estimate the useful life of a part to predict the end of its useful life. The limitations of this approach are widely recognized and range from the consideration that all products have the same life cycle curve, and the amount of business information needed to give a reasonable estimate. As shown previosly in Table 4, which summarizes the indexes and strategies developed over the years, It is important to highlight that each system requires or involves parameters related to commercial information, consumer behaviors, and technical product characteristics. In other words, trained personnel capable of handling this information are necessary to estimate the risk of product obsolescence. Although these methods allow for determining the risk of a component becoming obsolete during its life cycle, they do not provide information about the type of obsolescence it will present, representing a significant research gap in methodological design approaches. Without identifying the type or types of obsolescence a system faces, it is impossible to determine which design strategy is the most appropriate to mitigate it. In addition, other finding reveals that most research on obsolescence assessment strongly focuses on electronic products, emphasizing the need to extend these tools to other types of products.

Finally, strategic obsolescence management involves a broader, long-term approach that requires proactive planning and strategic decision-making to address obsolescence throughout the product or system lifecycle. While this strategy is becoming more widely known, it is still one of the least used. To implement this type of management, all information concerning the product is essential to plan its behavior throughout its lifecycle. However, obtaining this information can be a barrier since it is often unavailable or difficult to estimate. Technological advances can quickly make planned components and systems obsolete, requiring constant updates and constantly challenging obsolescence management. Despite its potential advantages, implementing obsolescence management strategies has several limitations: lack of data, lack of resources on the manufacturer's part, lack of priority, and lack of compatibility related to technological advances. There are also challenges in managing product family obsolescence, including the cost and complexity of replacing obsolete components and balancing the costs and benefits of platform replacement. In this regard, little literature has been found on product family obsolescence and how to manage it from the early stages of design.

### 4.4 Implications for management science/environmental and societal perspectives

From a managerial perspective Product obsolescence means products lose value faster, affecting how companies manage their stock. Deciding how much to ship becomes tricky when a company produces products at a rate close to costumer demand. Merging products with services can help companies earn more and combat obsolescence issues. When companies consider bundling products and services sustainably way, it can lead to better business models and encourage eco-friendly choices [10].

From the societal perspective, obsolescence is affected by producers, marketers, and even us as customers. Companies often update their products to keep ahead of competitors. However, frequent updates can make older products seem outdated or even “out of fashion” [74]. There’s also a common belief: if a product is cheap, it might not last long [75]. This affects how people buy and how much they value a product. Also, customers have a part to play; taking good care of their products can make them last longer.

In terms of environmental issues, obsolescence (especially planned) is getting a bad reputation because it's not eco-friendly. Some even see it as a business-related environmental issue because it results in more waste and frequent replacements [32]. Companies need to think long-term about how their products impact the environment to fix this. This starts with management. They should guide their teams in designing longer-lasting products and making better decisions for the environment. Tools and methods that consider how easy products are to use, adapt, and modify can also help [11]. Lastly, one suggestion is to have laws that set a minimum lifespan for products to ensure they last longer [32].

## 5 Conclusion

The accelerated production and consumption of new products and the disposal of older ones raise the need to address obsolescence from the earliest design stages. This review has aimed to highlight the current design methodologies and their relationship to obsolescence, identify the most relevant product design attributes that impact product obsolescence, and identify existing metrics or indicators to measure product obsolescence. Also, the results of this scoping review allowed us to provide guidance and recommendations on addressing obsolescence from early design phases.

A presentation on the evolution of obsolescence and a thematic analysis served as the basis for identifying the relationship of obsolescence with product design. Our findings reveal two design streams: designing products for long life and designing for life extension. While these methodologies focus on resisting and delaying obsolescence, there is no formal identification of product design attributes related to the different types of obsolescence, such as technological, functional, psychological, economic, DMSMS, and Planned. Additionally, we found that obsolescence has been managed reactively, proactively, and strategically, with metrics to calculate product obsolescence risk or identify critical components. However, these obsolescence forecasting metrics focus on defining whether a scenario occurs but do not consider what type of obsolescence the product may exhibit. Likewise, they depend on variables not included in the design stages, such as consumer behavior, commercial information, and technical characteristics. As a result, determining the most effective design strategy to reduce obsolescence can be time-consuming.

This work has reaffirmed that the obsolescence impact directly associated with a product's lifetime is given limited consideration in product development. Designers, producers, consumers, governments, businesses, and service providers are responsible for minimizing the impact of products on the environment throughout their lifetime. Therefore, some suggestions for future research are that more attention should be given to I) developing additional approaches focused on determining the definition of requirements and design attributes that impact product obsolescence (promoting or delaying), ii) Developing approaches aimed at defining/selecting materials to achieve product durability from the early design stages considering the product life cycle, iii) Developing more design methods and indicators to measure, determine and predict product obsolescence in different lifecycle scenarios.

The analysis and discussion of results were developed from an engineering point of view. However, it is noteworthy that addressing product obsolescence requires a multidisciplinary approach encompassing research areas such as consumer behavior and product aesthetics. As a result, this study has limitations, including the potential for researcher bias to affect the systematization of the information. Additionally, more recent literature may not have been included due to the time spent on data analysis and review. Section 2 outlines the methodology adopted to mitigate these issues as much as possible.

## Funding

This research work was supported by Vicerrectoría de Investigación, Creación e Innovación, 10.13039/501100004245Universidad del Norte under contract UN-OJ-2022-57,010.

## Data availability statement

Data supporting the findings of this study are not available within the article or its supplementary materials. Data associated with this study has not been deposited into a publicly available repository. Data will be made available on request.

## CRediT authorship contribution statement

**Lesly Sierra-Fontalvo:** Writing – original draft, Visualization, Validation, Investigation, Data curation. **Arturo Gonzalez-Quiroga:** Writing – review & editing, Supervision, Resources, Methodology, Funding acquisition, Formal analysis, Conceptualization. **Jaime A. Mesa:** Writing – review & editing, Writing – original draft, Validation, Supervision, Resources, Project administration, Methodology, Investigation, Funding acquisition, Data curation, Conceptualization.

## Declaration of competing interest

The authors declare the following financial interests/personal relationships which may be considered as potential competing interests:Lesly Sierra-Fontalvo financial support was provided by 10.13039/501100004245Universidad del Norte.
